# Multicentre Randomised trial of Acute Stroke treatment in the Ambulance with a nitroglycerin Patch (MR ASAP): study protocol for a randomised controlled trial

**DOI:** 10.1186/s13063-019-3419-z

**Published:** 2019-06-26

**Authors:** Sophie A. van den Berg, Diederik W. J. Dippel, Jeannette Hofmeijer, Puck S. S. Fransen, Klaartje Caminada, Arjen Siegers, Nyika D. Kruyt, Henk Kerkhoff, Frank-Erik de Leeuw, Paul J. Nederkoorn, H. Bart van der Worp, on behalf of the MR ASAP Investigators

**Affiliations:** 1 Department of Neurology, Amsterdam University Medical Center, University of Amsterdam, Meibergdreef 9, 1105 AZ Amsterdam, The Netherlands; 2 Department of Neurology, Erasmus MC University Medical Center, Doctor Molewaterplein 40, 3015 GD Rotterdam, The Netherlands; 3 Department of Neurology, Rijnstate, Wagnerlaan 55, 6815 AD Arnhem, The Netherlands; 4 Department of Neurology, Isala, Dokter van Heesweg 2, 8025 AB Zwolle, The Netherlands; 5 Regional Ambulance Service IJsselland, Voltastraat 3-A, 8013 PM Zwolle, The Netherlands; 6 Department of Emergency Medicine, Isala, Dokter van Heesweg 2, 8025 AB Zwolle, The Netherlands; 7 Ambulance Amsterdam, Karperweg 19-25, 1075 LB Amsterdam, The Netherlands; 8 Department of Anaesthesiology, Amsterdam University Medical Center, University of Amsterdam, Meibergdreef 9, 1105 AZ Amsterdam, The Netherlands; 9 Department of Neurology, Leiden University Medical Center, Albinusdreef 2, 2333 ZA Leiden, The Netherlands; 10 Department of Neurology, Albert Schweitzer Hospital, Albert Schweitzerplaats 25, 3318 AT Dordrecht, The Netherlands; 11 Department of Neurology, Radboud University Medical Center, Geert Grooteplein Zuid 10, 6525 GA Nijmegen, The Netherlands; 12 Department of Neurology and Neurosurgery, Brain Center University Medical Center Utrecht, Utrecht University, Heidelberglaan 100, 3584 CX Utrecht, The Netherlands

**Keywords:** Acute stroke, Glyceryl trinitrate, GTN, Nitroglycerin, Ambulance, Cerebrovascular disorders, Prehospital, Randomised controlled trial

## Abstract

**Background:**

Some studies have suggested that transdermal administration of glyceryl trinitrate (GTN; nitroglycerin) in the first few hours after symptom onset increases the chance of a favourable outcome after ischaemic stroke or intracerebral haemorrhage, possibly through an increase in intracranial collateral blood flow and a reduction in blood pressure. The Multicentre Randomised trial of Acute Stroke treatment in the Ambulance with a nitroglycerin Patch (MR ASAP) aims to assess the effect of transdermal GTN, started within 3 h after stroke onset in the prehospital setting, on functional outcome at 90 days in patients with acute ischaemic stroke or intracerebral haemorrhage.

**Methods:**

MR ASAP is a phase III, multicentre, randomised, open-label clinical trial with a blinded outcome assessment. A total of 1400 adult patients with suspected stroke and a systolic blood pressure ≥ 140 mmHg will be randomised to transdermal GTN (5 mg/day), administered as a transdermal patch by paramedics in the prehospital setting within 3 h of stroke onset and continued for 24 h or to standard care. The primary outcome is the score on the modified Rankin Scale (mRS) at 90 days, analysed with ordinal logistic regression. Secondary outcomes include blood pressure and collateral circulation at hospital admission, neurological deficit measured with the National Institutes of Health Stroke Scale at 24 h, and mortality and poor outcome (mRS score 3 to 6) at 90 days. This trial will be conducted in the Netherlands and will use a deferred consent procedure. The trial is part of the Collaboration for New Treatments of Acute Stroke (CONTRAST) programme.

**Discussion:**

MR ASAP will assess whether very early administration of GTN improves outcome after stroke in a setting where rates of intravenous thrombolysis and endovascular treatment for acute ischaemic stroke are high. The deferred consent procedure facilitates prompt GTN treatment and will prevent delay to revascularisation therapies. If early transdermal GTN treatment proves to be effective, this low-cost treatment can be readily implemented into daily clinical practice.

**Trial registration:**

ISRCTN Registry, ISRCTN99503308. Registered on 2 January 2018.

**Electronic supplementary material:**

The online version of this article (10.1186/s13063-019-3419-z) contains supplementary material, which is available to authorized users.

## Background

Despite recent advances in the treatment of patients with acute ischaemic stroke or intracerebral haemorrhage, about half of the patients still have a poor outcome [1]. Treatments with the largest benefit—intravenous thrombolysis and endovascular treatment—are indicated for a relatively small proportion of patients. Hence, there is a need for additional treatments applicable to a large range of patients with acute stroke.

Potential therapeutic targets in patients with acute stroke include improvement of collateral blood flow, in case of cerebral ischaemia, and reduction of blood pressure. High admission blood pressure in patients with ischaemic stroke or intracerebral haemorrhage has consistently been associated with poor outcome and is an easily modifiable factor [2].

In patients with acute intracerebral haemorrhage, rapid blood pressure lowering may be effective and is recommended in international guidelines [3, 4]. In patients with acute ischaemic stroke, the benefit of acute blood pressure lowering is even less certain [5]. After occlusion of an intracranial artery, blood supply to the potentially salvageable penumbra is dependent on collateral arteries, which can be defined as pre-existent artery-to-artery anastomoses that can provide blood to brain tissue when the primary supply pathways fail [6]. A systemic blood pressure reduction could lead to decreased blood supply to the ischaemic territory and increased ischaemic damage because of impaired cerebral autoregulation. By contrast, augmented collateral blood flow to the penumbra before recanalisation could reduce the extent of irreversible damage and might lead to a better functional outcome [7]. In addition, the chance of a favourable outcome after endovascular treatment is greatest in patients with good collateral blood flow and much smaller in patients with absent or poor collaterals [6, 8]. Therefore, optimising collateral perfusion may improve outcome after intravenous thrombolysis or endovascular treatment. Nitric oxide donors are candidate drugs for augmentation of collateral blood flow and blood pressure reduction by their vasodilatory effects [9]. In a meta-analysis of animal studies modelling acute ischaemic stroke, nitric oxide donors increased cerebral blood flow and reduced infarct size [10].

Glyceryl trinitrate (GTN), or nitroglycerin, is a nitric oxide donor and a systemic and cerebral vasodilator. The effects of transdermal administration of GTN have been tested in six randomised clinical trials in patients with acute ischaemic stroke or intracerebral haemorrhage [11–16]. Of these trials, the Efficacy of Nitric Oxide in Stroke (ENOS) trial was the largest, with 4011 patients included within 48 h after stroke onset [12]. An individual data meta-analysis (*n* = 4197) of the first five trials did not show an effect on functional outcome in the overall patient population. However, in a predefined subgroup of 312 patients randomised within 6 h of stroke onset, GTN reduced the risk of a poor functional outcome and death in a time-dependent manner. Benefit was observed both in patients with ischaemic stroke and in those with intracerebral haemorrhage. Compared to controls, GTN reduced systolic blood pressure at 1–2 h by an average of 9.4 mmHg [11].

Of the abovementioned 312 patients, 41 were enrolled in the ambulance-based phase II Rapid Intervention with Glyceryl Trinitrate in Hypertensive Stroke (RIGHT) trial. This trial assessed the feasibility and safety of prehospital treatment with GTN, started within 4 h of stroke onset [13]. Early administration of GTN lowered systolic blood pressure by 21 mmHg at 15 min after randomisation and by 18 mmHg at 2 h compared with controls, but was safe and associated with improved functional outcome.

The phase III RIGHT-2 trial assessed the safety and efficacy of transdermal GTN, started within 4 h of symptom onset and before admission to the hospital, in 1149 patients with presumed stroke. The results of this trial were published one year after the Multicentre Randomised trial of Acute Stroke treatment in the Ambulance with a nitroglycerin Patch (MR ASAP) had started. In RIGHT-2, very early administration of transdermal GTN did not improve functional outcome at 90 days [17].

MR ASAP aims to assess the effect of transdermal GTN, started within 3 h of symptom onset in the prehospital setting, on functional outcome at 90 days in patients with acute ischaemic stroke or intracerebral haemorrhage. Secondary objectives are to assess whether the effects of transdermal GTN on functional outcome are consistent across specific subgroups of patients, i.e. those with: (1) ischaemic stroke; (2) ischaemic stroke treated with endovascular treatment; or (3) intracerebral haemorrhage; and to assess the effects of GTN on collaterals, size of the ischaemic core and the amount of salvageable brain tissue on admission to the hospital.

## Methods/design

### Study design

**Fig. 1:**
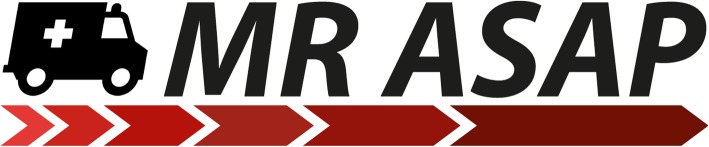
Trial logo

MR ASAP is a phase III multicentre clinical trial with randomised treatment allocation, open-label treatment and blinded endpoint assessment (PROBE) (Fig. 1). In this trial, prehospital treatment with transdermal GTN in a dose of 5 mg/day for one day will be compared with standard care, in 1400 (2 × 700) patients with suspected stroke.

### Patient population

The study population will consist of patients aged ≥ 18 years in the prehospital setting, in whom the attending paramedic diagnoses the patient with probable stroke with a moderately severe to severe deficit. Inclusion criteria are listed in Table 1 and exclusion criteria in Table 2. Several exclusion criteria are labelled as ‘known’, indicating that the patient should be excluded from participation in the trial only if the paramedic is aware of the presence of an exclusion criterion.

**Table 1:** Inclusion criteria

Age ≥ 18 years	
Probable diagnosis of acute stroke, as assessed by the paramedic in the prehospital setting	
Score of 2 or 3 on the Face-Arm-Speech Test (FAST)	
Systolic blood pressure ≥ 140 mmHg	
Possibility to start the trial treatment within 3 h of symptom onset	
Intention to transport the patient to one of the participating hospitals	
Written informed consent (deferred)

**Table 2:** Exclusion criteria

Considerable pre-stroke dependency in activities of daily living, defined as staying in a chronic nursing home or rehabilitation centre	
Known pregnancy or lactation	
Indication for acute treatment with nitroglycerin or known use of nitroglycerin in the previous 12 h	
Known hypersensitivity to GTN, nitrates in general or adhesives used in the patch	
Glasgow Coma Scale < 8	
Known with any of the following heart disorders: myocardial insufficiency due to obstruction; aortic or mitral valve stenosis; constrictive pericarditis; hypertrophic obstructive cardiomyopathy; cardiac tamponade	
Known marked anaemia, defined as haemoglobin < 5 mmol/L	
Known closed angle glaucoma	
Known use of phosphodiesterase type-5 inhibitors (e.g. sildenafil)	

*mRS* modified Rankin scale, *GTN* glyceryl trinitrate

### Randomisation and treatment

**Fig. 2:**
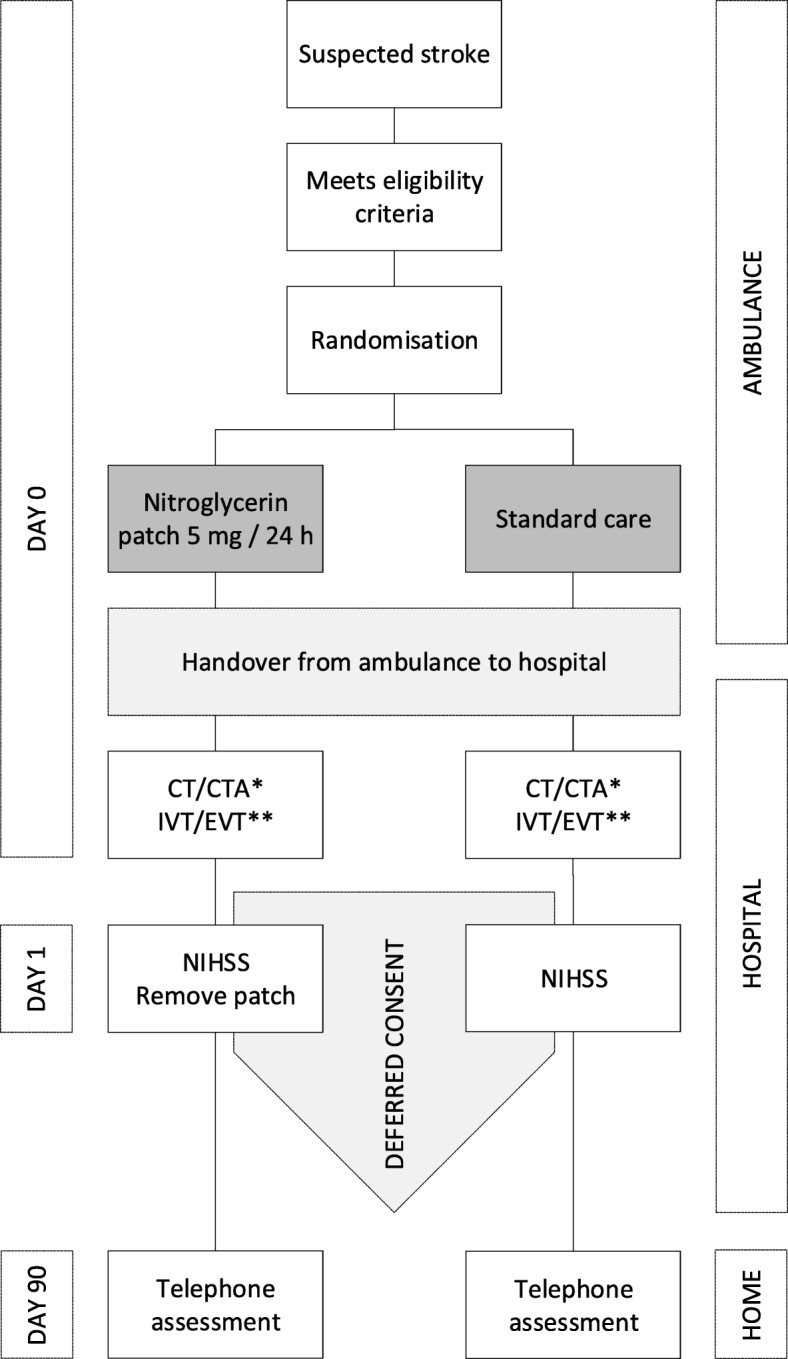
Flow of patients in the MR ASAP trial. Adapted from Appleton JP, Scutt P, Dixon M, Howard H, Haywood L, Havard D, et al. Ambulance-delivered transdermal glyceryl trinitrate versus sham for ultra-acute stroke: Rationale, design and protocol for the Rapid Intervention with Glyceryl trinitrate in Hypertensive stroke Trial-2 (RIGHT-2) trial (ISRCTN26986053). Int J Stroke. 2019;14:191–206. 10.1177/1747493017724627. *Performed as part of routine care. **If eligible and as part of routine care or within subsequent CONTRAST trial. CONTRAST Collaboration for New Treatments of Acute Stroke, CT computed tomography, CTA computed tomography angiography, EVT endovascular treatment, IVT intravenous thrombolysis, NIHSS National Institutes of Health Stroke Scale

Patients will be randomised 1:1 to open-label GTN (5 mg/day for one day) administered as a transdermal patch by paramedics in the prehospital setting within 3 h of stroke onset in addition to standard care, or to standard care alone (Fig. 2; Additional file 1: Figure S1). Trained paramedics will randomise patients through a secure web-based electronic application with real-time data validation, using random block sizes without strata. If patients are randomised to treatment with GTN but prove not to have a transient ischaemic attack, ischaemic stroke or intracerebral haemorrhage after examination in the hospital, the patch will be removed but patients will be followed-up as part of this study. If during the course of the first 24 h it becomes apparent that the patient did not fulfil the inclusion or exclusion criteria, the patch will also be removed. In case of hospital discharge before 24 h, the patch will be removed upon discharge. Otherwise, the nitroglycerin patch will be removed at 24 h. The patch will be removed by trained study personnel upon instruction of a local investigator.

Patients in each study group will be treated according to national and international guidelines and local protocols of ischaemic stroke, intracerebral haemorrhage or any other condition that may be diagnosed. Participation in this study does not preclude inclusion in a subsequent intervention study, as long as this is not directed at blood pressure modification.

### Outcomes

The main study outcome is functional outcome, assessed with the modified Rankin Scale (mRS) at 90 ± 14 days [18]. Secondary outcomes include but are not limited to: collateral circulation assessed with computed tomography (CT) angiography and sizes of the infarct core and perfusion deficit on CT perfusion on admission; neurological deficit measured with the National Institutes of Health Stroke Scale at 24 h; mortality, poor functional outcome (mRS 3 to 6), disability assessed with the Barthel Index, [19] and quality of life assessed with the EuroQol-5D-5L [20] at 90 days; and home time [21] and patient location over the first 90 days.

Safety outcomes are serious adverse events; hypotension or hypertension requiring clinical intervention; and symptomatic intracerebral haemorrhage, scored according to the Heidelberg Bleeding Classification, [22] all in the first seven days or until discharge, if earlier.

### Study procedures

Baseline characteristics assessed by the paramedic will be included in the prehospital eCRF via a mobile device that is connected to the Internet. The system randomises the patient and also allocates a unique subject identification number, with sequential numbering per ambulance service. Blood pressure and pulse will be assessed at baseline in the prehospital setting and at hospital admission. Where assessed as part of routine clinical practice during hospital admission, blood pressure and pulse will be collected at hourly intervals in the first 6 h, at 2-h intervals between 6 h and 24 h, and at 24-h (± 4 h) intervals thereafter. Body temperature will be collected at hospital admission and at 24 ± 4 h. The score on the Face-Arm-Speech Test (FAST) will be assessed in the prehospital setting by the paramedic and the score on the NIHSS at hospital admission by the treating physician. The NIHSS will also be assessed at 24 ± 4 h after study inclusion. During hospital admission no ad-ditional imaging or laboratory tests are required, but routinely performed imaging will be collected. Serious adverse events will be registered until seven days or until discharge, if earlier.

At 90 ± 14 days the scores on the mRS, Barthel Index, EQ-5D-5L, home time and patient location will be assessed by trained research personnel who are not aware of the treatment allocation. Standardised reports of the telephone interview will be used for the assessment of outcome on the mRS at 90 days by a central outcome committee, blinded to the allocated treatment.

### Informed consent

This study evaluates a treatment initiated by paramedics as soon as possible after stroke onset. Since most patients with acute neurological deficits are not capable of decision-making, and to reduce treatment delays, MR ASAP uses a deferred informed consent procedure, in line with Dutch law [23]. This implicates that patients are included and may receive the GTN patch in the ambulance before consent is obtained. Written informed consent should be obtained as soon as reasonably possible. In patients randomised to GTN who decline to participate, study medication will be stopped immediately. Patients who do not provide consent or withdraw during the study will be asked explicitly if their data can be used in a coded, non-traceable manner, to be able to describe baseline and treatment characteristics for the great majority of patients. If the patient continues to lack decision-making capacity, a legal representative will be asked to provide consent.

### Data and Safety Monitoring Board

An independent Data and Safety Monitoring Board (DSMB) will oversee the safety of patients in the trial and the efficacy of the intervention under study. They will work in accordance with a dedicated charter and will follow processes recommended by the DAMOCLES statement. The DSMB is formed by a professor of vascular medicine, a vascular neurologist and a cardiovascular epidemiologist. An independent trial statistician will combine data on treatment allocation with the clinical data in order to report to the DSMB. With respect to safety, the DSMB will conduct unblinded interim analyses after every 100 patients have completed follow-up. With respect to efficacy, the DSMB will conduct interim analyses after 900 patients had their final follow-up. The DSMB may recommend early termination of the study because of safety if the rate of a poor functional outcome at 90 days is lower in the control group, *p* < 0.01 (nominal, two-sided); or if the death rate at 90 days is lower in the control group, *P* < 0.01 (nominal, two-sided); and because of efficacy if the 99.9% confidence interval for the difference between the mean mRS scores at 90 days excludes the null hypothesis, that is, the groups are significantly different at *p* < 0.001; or because of any other reason the DSMB will find relevant to the trial.

### Sample size estimates

MR ASAP is powered to detect a statistically significant shift in the distribution of the scores on the mRS at 90 days in the overall study population, assuming an effect that leads to a 7% absolute risk reduction (ARR) in poor functional outcome (mRS 3 to 6) in the GTN group compared with controls (48% vs 41%). The sample size calculations are based on a simulation study with a proportional odds model. The risk of a poor functional outcome of 48% in this domain of patients is based on findings in the Field Administration of Stroke Therapy – Magnesium (FAST-MAG) trial, a trial including 1700 patients with suspected stroke in the prehospital setting [24]. We assume that the use of covariate adjustment will increase statistical efficiency by 20%, which is based on the R^2^ (coefficient of determination) in the Multicenter Randomized Clinical Trial of Endovascular Treatment for Acute Ischemic Stroke in the Netherlands (MR CLEAN) data of an adjustment model containing age, pre-stroke mRS and items 4, 5a, 5b, 9 and 10 of the NIHSS that correspond to the FAST score [25, 26]. A total study size of 1280 patients allows for a power of 80% to detect a difference at a 5% significance level in the scores on the mRS in patients treated with GTN compared to controls. As we expect to include around 10% stroke mimics, in whom no treatment benefit is expected, we increased this sample size with 10% and aim for inclusion of 1400 patients.

### Statistical analyses

The primary effect estimate, which is the shift in the mRS score at 90 days, will be assessed by means of ordinal logistic regression and expressed as a common odds ratio with a 95% confidence interval. The statistical analyses will be performed according to the intention-to-treat principle and will therefore include patients with a stroke mimic. Analyses will be adjusted for relevant baseline characteristics including age, sex, stroke type, score on the FAST at study inclusion, time from symptom onset to randomisation, pre-stroke score on the mRS and ambulance region. Before follow-up is completed, a final statistical analysis plan will be developed that specifies the hypotheses to be tested and a description of treatment effects, statistical methodology and subgroup analyses in detail.

### Study organisation

MR ASAP is conducted by members of the Collaboration for New Treatments of Acute Stroke (CONTRAST) and investigators in the participating ambulance services and hospitals. The CONTRAST consortium performs five large randomised clinical trials in acute stroke patients in the Netherlands to test novel treatment strategies (Table 3). Patients enrolled in the MR ASAP trial can also participate in one of the subsequent trials within the CONTRAST consortium (https://www.contrast-consortium.nl) [27].

**Table 3:** Randomised trials within the CONTRAST consortium

Multicentre Randomised trial of Acute Stroke treatment in the Ambulance with a nitroglycerin Patch (MR ASAP): pre-hospital augmentation of collateral blood flow and blood pressure reduction (ISRCTN99503308)	
Multicentre Randomised CLinical trial of Endovascular treatment for Acute ischaemic stroke in the Netherlands. The effect of concomitant MEDication: heparin, antiplatelet agents, both or neither (MR CLEAN-MED): antithrombotics to prevent microvascular occlusion after EVT (ISRCTN76741621)	
Intravenous treatment followed by intra-arterial treatment versus direct intra-arterial treatment for acute ischaemic stroke caused by a proximal intracranial occlusion (MR CLEAN-NO IV): immediate EVT without preceding thrombolysis (ISRCTN80619088)	
Multicentre Randomised Clinical Trial of Endovascular Stroke treatment in The Netherlands for Late arrivals: (MR CLEAN-LATE): EVT in the 6 to 24 h time window (ISRCTN19922220)	
The Dutch ICH Surgery Trial pilot study (DIST pilot study): minimally invasive endoscopy-guided surgery for spontaneous intracerebral haemorrhage (NCT03608423)	

*CONTRAST* Collaboration for New Treatments of Acute Stroke, *EVT* endovascular treatment, *ICH* intracerebral haemorrhage

The sponsor of the trial is University Medical Center Utrecht. The trial is led by two co-chief investigators (stroke neurologists at the University Medical Center Utrecht and Amsterdam University Medical Center, respectively), a coordinating investigator and a trial executive committee that consists of eight neurologists and two ambulance service physicians. The writing committee will also be based on the trial executive committee. Each participating centre, including each participating ambulance service, has a local principal investigator. Publications will be made on behalf of all MR ASAP investigators (https://www.mrasap.nl) [28]. We plan to publish the main results in a peer-reviewed journal, with authorship contributions according to the International Committee of Medical Journal Editors (ICMJE) criteria.

All data will be entered into a web-based trial management system that allows for edit and audit trials and has range and date checks, by trained local physicians or research nurses. Incoming data will be reviewed by the central trial office. Local data will be carefully monitored by checking the first three and thereafter a sample of 10% patient case report forms against source data. Within the CONTRAST consortium, subcommittees will be composed for outcome assessment, adverse event adjudication and imaging assessment.

## Discussion

MR ASAP assesses the effect of transdermal GTN, started within 3 h after stroke onset in the prehospital setting, on functional outcome at 90 days in patients with acute ischaemic stroke or intracerebral haemorrhage. Previous trials of acute stroke treatment started by paramedics, FAST-MAG (United States), RIGHT and RIGHT-2 (United Kingdom), have shown that enrolment and very early treatment of acute stroke patients in the prehospital setting is feasible [13, 17, 24]. The median time intervals from symptom onset to study drug administration were 45, 55 and 70 min, respectively.

For patients with ischaemic stroke, timely restoration of blood flow is a first priority. In randomised trials, intravenous thrombolysis with alteplase and endovascular treatment have shown to increase the chance of a favourable outcome in selected patients, [1, 29] with earlier treatment associated with greater benefit [30]. However, in meta-analyses of these trials, about half of the patients were dead or dependent at three months [1, 29]. One explanation for the lack of benefit in a considerable number of patients is that much of the ischaemic brain tissue may have sustained irreversible damage at the time that the occluded artery is recanalised. Improved collateral blood flow to the penumbra before the start of intravenous thrombolysis or endovascular treatment could reduce the extent of irreversible damage and might lead to a better functional outcome [7]. In animal studies of focal cerebral ischaemia, NO donors increased cerebral blood flow and reduced infarct size, [10] but the effects of GTN on collateral flow in patients with ischaemic stroke are still uncertain. These effects will be assessed in MR ASAP with CT angiography and CT perfusion on admission to the hospital.

MR ASAP will include patients within 3 h of the onset of symptoms suggestive of acute stroke. This very short time window was selected because we assumed a greater beneficial effect of transdermal GTN the sooner treatment is started, as suggested in a pooled analysis of previous trials of GTN in patients with acute stroke, [11, 31] but not in RIGHT-2 [17]. Such very early treatment is considered feasible in the majority of stroke patients in the Netherlands. A cohort study of patients treated with intravenous thrombolysis in a comprehensive stroke centre in the Dutch capital showed a median onset-to-door time of 71 min [32]. With the exception of a short physical examination, no diagnostic work-up needs to be performed before inclusion in this study; therefore, rapid inclusion is possible.

For safety reasons, only patients with a systolic blood pressure of ≥ 140 mmHg are eligible for inclusion in this study. In RIGHT, systolic blood pressure at 15 min after randomisation was 21 mmHg lower in patients treated with GTN than in controls, without an increase in serious adverse events [13]. In the subgroup of patients treated within 6 h of stroke onset in the above-mentioned pooled analysis, GTN resulted in a 10 mmHg lower systolic blood pressure at 1–2 h than standard treatment. In the complete study population, 2.7% of the patients treated with GTN required treatment for clinical hypotension compared to 0.7% of controls, but no increase in the rates of serious adverse events or death was observed [11]. In RIGHT-2, GTN reduced systolic blood pressure by 5.8 mmHg at hospital admission compared with the control group. The complication rate was similar for both groups [17]. GTN has also been shown safe in patients with ipsilateral carotid stenosis [12].

The informed consent procedure in acute stroke research is challenging. Besides fast diagnostic work-up and initiation of time-dependent treatments in a person with neurological deficits, informed consent must be obtained from a vulnerable group of patients who often lack decision-making capacity. In the Netherlands, deferred informed consent is possible in emergency situations as long as circumstances prevent the provision of informed consent and if inclusion in the trial may benefit the person in urgent need of medical treatment [23].

For MR ASAP, we considered deferred consent justifiable and this has been approved by the Research Ethics Committee. First, inclusion in this prehospital study should not delay transportation to the hospital and reduce the possibility to receive effective treatments such as intravenous thrombolysis or endovascular treatment. Second, participation in the trial may be of direct benefit to the patient in a time-dependent manner [11]. Third, not only the very large majority of patients will lack decision-making capacity due to their neurological symptoms, but also most relatives can be considered not capable of providing consent due to stress and the necessity of urgent treatment. Also, relatives are often not available in the very acute setting. In addition, GTN was safe in previous trials of acute stroke, with no difference in total SAEs and death rates in patients treated with GTN as compared with controls [11].

Since patients are enrolled before examination by a stroke specialist and imaging, we have selected a score of 2 or 3 on the FAST in addition to the clinical assessment of a trained paramedic in order to keep the proportion of patients with stroke-mimicking conditions as small as possible. The FAST test is widely used by paramedics in The Netherlands and yields a positive predictive value for true strokes up to 89% [33]. The FAST-MAG trial and RIGHT trial have shown that the proportion of enrolled patients with stroke-mimicking conditions can be as small as 4–12%. We have accounted for 10% stroke mimics in the sample size calculation of MR ASAP for whom no harm of GTN treatment is expected.

The RIGHT-2 trial has recently investigated the effects of prehospital stroke treatment with a GTN patch in the United Kingdom [34]. Similar to MR ASAP, in RIGHT-2 the paramedic assessed the eligibility criteria for enrolment in the trial and starts study treatment. Likewise, RIGHT-2 used a FAST score of 2 or 3 as an inclusion criterion. In contrast to MR ASAP, patients were enrolled with a systolic blood pressure ≥ 120 mmHg. MR ASAP will be performed in a setting where approximately 20% of patients with acute ischaemic stroke receive intravenous thrombolysis and 5% endovascular treatment [35]. While informed consent is deferred in MR ASAP, in RIGHT-2 paramedics had to assess decision-making capacity (Additional file 2: Table S1). After completion of the trials, data of RIGHT-2 and MR ASAP will be pooled to improve the accuracy of estimates of the effect size.

In summary, improvement of intracranial collateral circulation and blood pressure reduction are potential targets for acute stroke treatment. MR ASAP assesses the effects of prehospital treatment with a GTN patch in suspected stroke. A deferred consent procedure is used to reduce delays in transport and treatment in the hospital. In case of effectiveness of GTN, the pragmatic design of the trial will simplify the implementation of GTN treatment in clinical practice Additional file 4.

### Trial status

Currently four ambulance services are enrolling patients in MR ASAP with possible referral to 14 participating hospitals. The first patient was included on 4 April 2018. By February 2019, 180 patients had been included. Recruitment by at least four other ambulance services will commence in 2019. Final follow-up is expected in 2021. The current article is based on protocol version 1.5 (December 2018).

After the presentation and publication of the results of RIGHT-2, inclusion in the trial was interrupted temporarily to allow DSMB review of all data from previous trials and safety and outcome date of the first 100 patients included in MR ASAP. This interim analysis was performed on 30 April 2019 and based on the advice of the DSMB, the trial will be restarted in July 2019.

## Additional files

Additional file 1: **Figure S1**. Schedule of enrolment, interventions and assessments. (PDF 128 kb)

Additional file 2: **Table S1.** Comparison of MR ASAP trial with RIGHT-2 trial. (PDF 29 kb)

Additional file 3: List of CONTRAST work package leaders. (PDF 31 kb)

Additional file 4: SPIRIT 2013 Checklist: Recommended items to address in a clinical trial protocol and related documents. (PDF 58 kb)

## Data Availability

The datasets of the current study will become available from the corresponding author on reasonable request, after publication of the main results.

## Abbreviations

CT: Computed tomography
CTA: Computed tomography angiography
DSMB: Data and Safety Monitoring Board
FAST: Face-Arm-Speech Test
GTN: Glyceryl trinitrate
IVT: Intravenous thrombolysis
mRS: Modified Rankin Scale
NIHSS: National Institutes of Health Stroke Scale
