# Supplementary material for: Multicentre Randomised trial of Acute Stroke treatment in the Ambulance with a nitroglycerin Patch (MR ASAP): study protocol for a randomised controlled trial
Source: Trials. 2019 Jun 26;20:383. doi: 10.1186/s13063-019-3419-z (PMC6595565; doi:10.1186/s13063-019-3419-z)
Supplement: Supplementary file 1 — Figure S1. Schedule of enrolment, interventions and assessments. (PDF 128 kb) [file 13063_2019_3419_MOESM1_ESM.pdf]

Supplementary Figure 1. Schedule of enrolment, interventions and assessments

	**STUDY PERIOD**				
**TIMEPOINT**	**0**	***1***	***3***	***5-7***	***90***
**ENROLMENT:**					
Eligibility screen	X				
Allocation	X				
Informed consent	◆—	—	→		
**INTERVENTIONS:**					
Nitroglycerin patch	◆—	◆			
**ASSESSMENTS:**					
Stroke severity (NIHSS)	X	X			
Blood pressure	◆—	—	—	◆	
modified Rankin Scale				X	X
Barthel Index, EuroQol-5D-5L					X
Reporting of SAEs	◆—	—	—	◆	

NIHSS: National Institutes of Health Stroke Scale; SAEs: Serious Adverse Events
