# Supplementary material for: Multicentre Randomised trial of Acute Stroke treatment in the Ambulance with a nitroglycerin Patch (MR ASAP): study protocol for a randomised controlled trial
Source: Trials. 2019 Jun 26;20:383. doi: 10.1186/s13063-019-3419-z (PMC6595565; doi:10.1186/s13063-019-3419-z)
Supplement: Supplementary file 2 — Table S1. Comparison of MR ASAP trial with RIGHT-2 trial. (PDF 29 kb) [file 13063_2019_3419_MOESM2_ESM.pdf]

Supplementary Table 1. Comparison of MR ASAP trial with RIGHT-2 trial

**Criteria**	**MR ASAP**	**RIGHT-2**
Design	Open-label, blinded endpoint, multicentre	Sham, blinded endpoint, multicentre
Screen for eligibility	Paramedic	Paramedic
Treatment initiation	Paramedic	Paramedic
Informed consent	Deferred informed consent in hospital by patient or proxy	Informed consent by paramedic by patient, or proxy consent by relative/paramedic
Eligibility criteria		
Age	*≥ 18*	*≥ 18*
FAST	*2 or 3*	*2 or 3*
SBP	*≥ 140 mm Hg*	*≥ 120 mm Hg*
Time to treatment	*< 3 hours*	*< 4 hours*
GCS	*8-15*	*8-15*
Randomisation	Electronic device	Identical treatment packs
GTN patch brand	Deponit-T	Transiderm-Nitro
Dose	5 mg / 24 hours	5 mg / 24 hours
Duration of treatment	24 hours	4 days
Sample size	1400	850
Primary outcome	90 day mRS, ordinal regression	90 day mRS, ordinal regression

FAST: Face Arm Speech Test; GCS: Glasgow Coma Scale; GTN: Glyceryl Trinitrate; mRS: modified Rankin Scale; MR ASAP: Multicentre Randomised trial of Acute Stroke treatment in the Ambulance with a nitroglycerin Patch; RIGHT-2: Rapid Intervention With Glyceryl Trinitrate in Hypertensive Stroke-2; SBP: systolic blood pressure
