# Supplementary material for: Multicentre Randomised trial of Acute Stroke treatment in the Ambulance with a nitroglycerin Patch (MR ASAP): study protocol for a randomised controlled trial
Source: Trials. 2019 Jun 26;20:383. doi: 10.1186/s13063-019-3419-z (PMC6595565; doi:10.1186/s13063-019-3419-z)
Supplement: Supplementary file 3 — List of CONTRAST work package leaders. (PDF 31 kb) [file 13063_2019_3419_MOESM3_ESM.pdf]

Additional File 3: List of CONTRAST work package leaders

**Principal Investigator**	**Organisation**
Beusekom, Heleen van, PhD	Erasmus MC Experimental Cardiology
Buskens, Erik, MD PhD	UMCG Epidemiology
Cate, Hugo ten, MD PhD	MUMC Thrombosis Expertise Center
Dammers, Ruben, MD PhD	Erasmus MC Neurosurgery
Dippel, Diederik MD PhD	Erasmus MC Neurology
Dijkhuizen, Rick, PhD	UMCU Center for Image Sciences
Kappelle, Jaap, MD PhD	UMCU Neurology
Klijn, Karin, MD PhD	UMCN Neurology
Koudstaal, Peter, MD PhD	Erasmus MC Neurology
Lingsma, Hester, PhD	Erasmus MC Public Health
Lugt, Aad van der, MD PhD	Erasmus MC Radiology
Luijckx, Gert Jan, MD PhD	UMCG Neurology
Maat, Moniek de, PhD	Erasmus MC Hematology
Majoie, Charles, MD PhD	Amsterdam UMC Radiology
Nederkoorn, Paul, MD PhD	Amsterdam UMC Neurology
Oostenbrugge, Robert van, MD PhD	MUMC Neurology
Roos, Yvo, MD PhD	Amsterdam UMC Neurology
Vivien, Denis, MD PhD	UNC INSERM
Worp, Bart van der, MD PhD	UMCU Neurology
Zwam, Wim van, MD PhD	MUMC Radiology
